# CRISPR/Cas9-mediated precise targeted mutagenesis of phytoene desaturase in celery

**DOI:** 10.1093/hr/uhac162

**Published:** 2022-08-01

**Authors:** Jie-Xia Liu, Tong Li, Hao Wang, Yan-Hua Liu, Kai Feng, Ao-Qi Duan, Hui Liu, Sheng Shu, Ai-Sheng Xiong

**Affiliations:** State Key Laboratory of Crop Genetics and Germplasm Enhancement, Ministry of Agriculture and Rural Affairs Key Laboratory of Biology and Germplasm Enhancement of Horticultural Crops in East China, College of Horticulture, Nanjing Agricultural University, 1 Weigang, Nanjing, 210095, China; State Key Laboratory of Crop Genetics and Germplasm Enhancement, Ministry of Agriculture and Rural Affairs Key Laboratory of Biology and Germplasm Enhancement of Horticultural Crops in East China, College of Horticulture, Nanjing Agricultural University, 1 Weigang, Nanjing, 210095, China; State Key Laboratory of Crop Genetics and Germplasm Enhancement, Ministry of Agriculture and Rural Affairs Key Laboratory of Biology and Germplasm Enhancement of Horticultural Crops in East China, College of Horticulture, Nanjing Agricultural University, 1 Weigang, Nanjing, 210095, China; State Key Laboratory of Crop Genetics and Germplasm Enhancement, Ministry of Agriculture and Rural Affairs Key Laboratory of Biology and Germplasm Enhancement of Horticultural Crops in East China, College of Horticulture, Nanjing Agricultural University, 1 Weigang, Nanjing, 210095, China; State Key Laboratory of Crop Genetics and Germplasm Enhancement, Ministry of Agriculture and Rural Affairs Key Laboratory of Biology and Germplasm Enhancement of Horticultural Crops in East China, College of Horticulture, Nanjing Agricultural University, 1 Weigang, Nanjing, 210095, China; State Key Laboratory of Crop Genetics and Germplasm Enhancement, Ministry of Agriculture and Rural Affairs Key Laboratory of Biology and Germplasm Enhancement of Horticultural Crops in East China, College of Horticulture, Nanjing Agricultural University, 1 Weigang, Nanjing, 210095, China; State Key Laboratory of Crop Genetics and Germplasm Enhancement, Ministry of Agriculture and Rural Affairs Key Laboratory of Biology and Germplasm Enhancement of Horticultural Crops in East China, College of Horticulture, Nanjing Agricultural University, 1 Weigang, Nanjing, 210095, China; State Key Laboratory of Crop Genetics and Germplasm Enhancement, Ministry of Agriculture and Rural Affairs Key Laboratory of Biology and Germplasm Enhancement of Horticultural Crops in East China, College of Horticulture, Nanjing Agricultural University, 1 Weigang, Nanjing, 210095, China; State Key Laboratory of Crop Genetics and Germplasm Enhancement, Ministry of Agriculture and Rural Affairs Key Laboratory of Biology and Germplasm Enhancement of Horticultural Crops in East China, College of Horticulture, Nanjing Agricultural University, 1 Weigang, Nanjing, 210095, China

Dear Editor

Celery (*Apium graveolens* L.) is a leafy vegetable crop of Apiaceae with economic importance, which is widely cultivated all over the world [1]. In production, improving quality, disease, and insect resistance, and late bolting are required for celery through traditional or modern molecular genetic improvement methods. Genetic improvement via conventional breeding was limited to the long breeding cycle and randomness; the necessity of genetic engineering breeding, therefore, has been highlighted. A precise genome-editing technology holds the potential to overcome the limitations of conventional breeding. Additionally, the research of functional genomics in celery also raised higher requirements for the development of genome-editing technology. An immature genetic transformation system and undeveloped gene-editing technology has become the bottleneck for basic research and genetic improvement in celery relative to other major crops.

The CRISPR/Cas9 system is an RNA-guided genome editing tool that consists of a Cas9 nuclease and a single-guide RNA (sgRNA) to generate efficient targeted modification [2, 3]. Due to its high efficiency and accuracy, CRISPR/Cas9-induced genome editing has been extensively applied in a variety of plant species, to improve plant resistance and yield and to study the function of genes in the control of agronomic traits [2–4]. Herein is constituted the first report of the successful establishment of a CRISPR/Cas9-based genome-editing system and validation of its efficacy by the targeted knockout of phytoene desaturase gene (*AgPDS*) in celery cv. ‘Jinnan Shiqin’. PDS, a rate-limiting enzyme in carotenoid biosynthesis, catalyzes the conversion of colorless phytoene into ζ-carotene, which further transformed into lycopene. It is commonly used as a visual marker to validate precise genome modification in a considerable number of species, as disruption of its function leads to albinism.

Celery is not readily transformable; there are few reports about the obtaining of celery transgenic plants. Development of a highly effective regeneration system is a pre-requisite for celery genetic transformation. We first sought to compare the efficacy of varied hormone combinations, and thus to find a suitable method for celery tissue culture and plant regeneration (see online supplementary Materials and Methods for more details). The hypocotyls of ‘Jinnan Shiqin’ seedlings were cut into segments (3–5 mm long) and used as explants. Prepared explants were cultivated on Gamborg B5 medium with different combinations of auxin (2,4-dichlorophenoxyacetic acid, 2,4-D) and cytokinin (kinetin, KT); the regeneration plantlets were obtained successfully (Fig. 1a). Calli induction rates exceeded 87% in celery explants grown on Gamborg B5 medium applied with 1.0–2.0 mg/L 2,4-D and 0.5–1.0 mg/L KT (see online supplementary Table S1). Under these conditions with the hormone ratio of 2:0.5 (2,4-D:KT), the calli induction rate and differentiation rate of ‘Jinnan Shiqin’ reached 95% and 78.5%, respectively, suggesting that this hormone combination was a suitable resource for celery genetic transformation.

**Figure 1:**
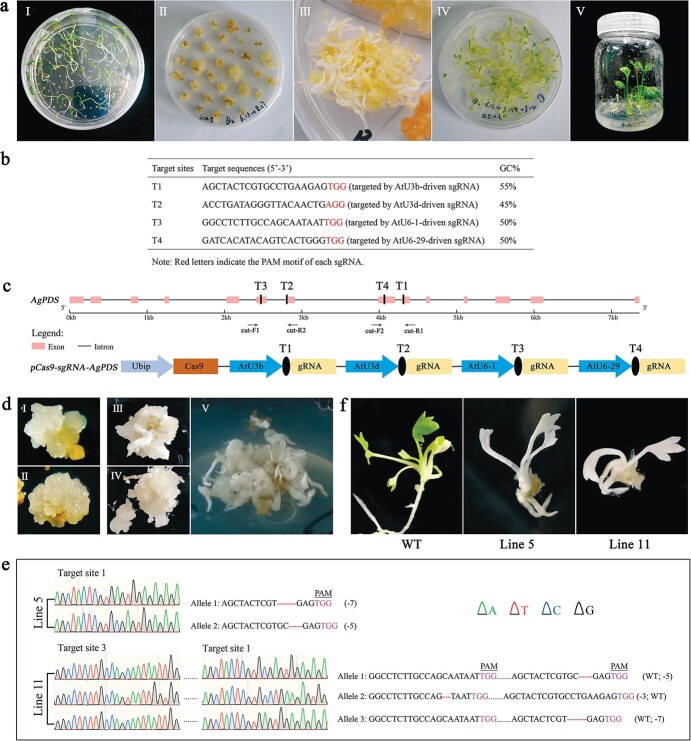
CRISPR/Cas9-mediated genome editing of celery targeting phytoene desaturase (*AgPDS*) gene. **a** Regeneration of ‘Jinnan Shiqin’ celery plants. **I**, Seedlings for tissue culture; **II**, Calli induced from celery explants; **III**–**IV**, Differentiated plants under light and dark conditions; **V**, Domestication. **b** The sequences of four target sites selected for *AgPDS*-editing. **c** Schematic map of CRISPR/Cas9 construct for the *AgPDS* editing. T1, T2, T3, and T4 represent the positions of the four target sites, respectively. Cut-F1/R2 and cut-F2/R1 flank two sgRNA target sites each and indicate binding sites of the primers used for PCR amplification. **d** Variegated calli produced by genetic transformation of *pCas9-sgRNA-AgPDS*, including yellowish (I, II) and white (III, IV, V) calli*.***e** Sequence analysis of two independent *AgPDS* mutant plants (lines 5 and 11). The sequencing chromatograms of mutated target site regions are shown. The nucleotide changes (dashes for deletion and WT for wild type) are also indicated on both sides of each sequence. Dots represent nucleotides that are not shown. The PAM sequence is highlighted in pink. **f** The phenotypes of *AgPDS* gene-editing mutants and wild-type plants without *AgPDS* gene-editing regenerated from kanamycin-resistant calli.

The genetic transformation technology and whole genome sequences, only recently available, made it possible to realize the potential of CRISPR-based genome editing in celery [5, 6]. The complete sequences of *AgPDS* CDS and genomic DNA in diploid celery were identified from the celery genome database [5, 6]. The annotation of AgPDS was validated using the BLASTP tool in the NCBI database. By PCR amplification and sequencing, the full length of *AgPDS* sequence was verified (see online supplementary Fig. S1). PDS is encoded by a single copy gene in celery, which has 14 exons and 13 introns. To construct the CRISPR/Cas9 plasmid for *AgPDS* gene-editing, target sites within the amplified *AgPDS* gene were designed using the online software CRISPR-GE [7]. The output four target sites of *AgPDS* that were located on the sixth, seventh, eighth, and ninth exons, respectively, were selected for designing sgRNA sequences (Fig. 1b). The four sgRNAs expression cassettes within the four target site sequences individually driven by AtU3b, AtU3d, AtU6-1, and AtU6-29 promotor were generated and inserted into a single 2300GN-Ubi-Cas9 binary vector [8] (a modified pYLCRISPR/Cas9Pubi-H vector [9]) between *Sbf* I and *Sma* I sites. The synthesized sgRNA along with the corresponding promoters and the 2300GN-Ubi-Cas9 map are listed in online supplementary Fig. S2. The Cas9 binary construct was built and designated as *pCas9-sgRNA-AgPDS* (Fig. 1c).

The construct expressing gRNA targeting the *AgPDS* gene was introduced into *Agrobacterium tumefaciens* strain GV3101, and then used to infect the ‘Jinnan Shiqin’ celery explants that pre-cultured on Gamborg B5 medium containing 2,4-D and KT. The detail of transformation and regeneration procedures are listed in the online supplementary Materials and Methods. After transformation with *pCas9-sgRNA-AgPDS*, explants produced yellowish or white calli (Fig. 1d). Regenerated celery plants obtained using the *Agrobacterium*-mediated transformation method showed phenotypes of green and albinism. Part of the albino plantlets did not form normal roots and look to be dwarf.

To validate the efficiency of the CRISPR/Cas9 system for targeted the editing of the *AgPDS* gene in celery, the genome DNA of albino plantlets generated from *AgPDS* gene editing were extracted and purified. The *Cas9* fragment was amplified by PCR to identify the putative gene editing plants (online supplementary Fig. S3). Albino plantlets without amplified *Cas9* fragment may be derived from the chloroplast genome aberration caused by somatic clonal variation *in vitro* culture or the large-scale deletions in the plastid genome interfering with chloroplast development [10]. The PCR-positive plants were subjected to detection of the mutation in the *AgPDS* genomic sequence. The genomic regions within four targets of *AgPDS* were amplified using two pairs of primers, cut-F1/R2 and cut-F2/R1 (online supplementary Table S2), to identify the mutation patterns by direct Sanger sequencing, yielding superimposed sequence chromatograms at two target sites (online supplementary Fig. S4). Then, the fragment of *AgPDS* containing four targets was cloned into the pGBKT7 vector and transformed into *Escherichia coli* (DH5α) followed by sequencing individual clones to decode superimposed sequence chromatograms from direct sequencing.

After experimental analysis and confirmation, two mutant lines were identified. The two obtained *AgPDS* gene-editing plants, line 5 and line 11, had deletion mutations occurring at target sites 1 and 3 (Fig. 1e). Sanger sequencing of line 5 transgenic plantlets showed a biallelic mutation and exhibited 5-bp or 7-bp deletions in the two strands of duplex DNA, respectively. Triallelic chimeric mutations occurred in line 11 plants, including 3-bp deletions in target site 3, and 5-bp and 7-bp deletions in target site 1. Based on the verification of subcloning, it is speculated that line 11 may be a chimera, in which the deletion of 3-bp in target site 3 accounts for a relatively low proportion, and the plant showed albinism in general. In two *AgPDS*-knockout transformants, the mutation events occurred within 4 to 10 bp upstream of the PAM sequences. The albino is one of the representative phenotypes frequently seen in the regenerated plantlets. There are few albino plants found in the plant regeneration process of celery. The albinism in plants without gene editing may be caused by the genome aberration related to chloroplast *in vitro* culture. Here, due to the mutation of the *AgPDS* gene in the celery genome, both lines 5 and 11 were manifested as albino plants (Fig. 1f). The current work proved that CRISPR/Cas9 is an effective approach and has a wide prospect for application in gene editing in celery plants.

## Acknowledgements

The research was supported by the Jiangsu seed industry revitalization project [JBGS (2021)068], the Key Research and Development Program of Jiangsu (BE2022386), the Key Research and Development Program of Suqian and Huaian (L202108, HAN202108) and the Priority Academic Program Development of Jiangsu Higher Education Institutions Project (PAPD).

## Author contributions

A.-S.X. and J.-X.L. initiated and designed the research, J.-X.L., H.W., K.F., T.L., A.-Q.D., H.L. and Y.-H.L. performed the experiments; J.-X.L., H.W., Y.-H.L. and S.S. analysed the data; A.-S.X. contributed reagents/materials/analysis tools; J.-X.L. wrote the paper; A.-S.X., J.-X.L. and T.L. revised the paper. All authors read and approved the final manuscript.

## Data availability

The data sets supporting the conclusions of this article are included within the article and the online supplementary data.

## Conflict of interest

The authors declare no conflict of interest.

## Supplementary data

Supplementary data is available at *Horticulture Research* online.

## Supplementary Material

Web_Material_uhac162
