# Supplementary material for: CRISPR/Cas9-mediated precise targeted mutagenesis of phytoene desaturase in celery
Source: Hortic Res. 2022 Aug 1;9:uhac162. doi: 10.1093/hr/uhac162 (PMC9531335; doi:10.1093/hr/uhac162)
Supplement: Web_Material_uhac162 [file web_material_uhac162.docx]

**Supplementary Document S1**

This document includes the following contents:

**Pages 2-5**: Supplementary Materials and Methods

**Page 5**: References for Supplementary Materials and Methods

**Pages 6-11**: Supplementary Figures

**Pages 12-13**: Supplementary Tables

**Supplementary Materials and Methods**

**Plant materials**

Seeds of celery cv. ‘Jinnan Shiqin’ were surface sterilized with 20% (v/v) sodium hypochlorite plus 0.02% (v/v) Tween 20 by continuous keeping for 30 min, then rinsed 3 times with sterile distilled water (ddH_2_O). The sterilized celery seeds were inoculated on MS (Murashige and Skoog, 1962) ^1^ solid medium (pH 5.8) for germination under low light conditions and the sterile seedlings were obtained.

**Calli induction and plant differentiation of celery**

The hypocotyls of celery sterile seedlings grown on MS solid medium for 10~12 days were cut into 3~5 mm segments and used as the explants. Obtained explants were placed on Gamborg B5 ^2^ calli induction medium supplemented with different proportions of plant hormones (2,4-D: KT = 0.5:0.6, 0.6:0.8, 0.5:1, 1:0.5, 1:1 and 2:0.5, mg/L) and cultured in dark at 25 ^o^C. After 60 days of induction culture, the calli induction rate was counted. Calli induction rate = (number of explants with calli generation / total number of explants) × 100%.

Secondly, the induced celery calli was transferred to Gamborg B5 solid medium without hormones to induce plant differentiation. After 30 days of culture, the differentiation rate of celery calli was counted. Calli differentiation rate = (number of differentiated calli / total number of calli) × 100%. According to the induction rate and differentiation rate of calli, the optimum regeneration conditions of celery tissue culture were summarized.

**Isolation, cloning, and sequence analysis of *AgPDS* in celery**

The whole genome sequences published recently make it possible to realize CRISPR/Cas9-based genome editing in celery ^3,4^. Annotated PDS sequences of carrot (DcPDS, XM_017396654.1), tobacco (NtPDS, XP_019244024.1), and maize (ZmPDS, NP_001338939.1) were used for BLAST analysis in celery genome database ^4,5^ to identify the sequence of homologous gene. The complete sequences of CDS and genomic DNA of a single-copy *AgPDS* were identified and functional annotated by BLASTP analysis in NCBI database. On the basis of the conserved sequence of *AgPDS* gene, a pair of primers (forward: 5’-TTTACAATTACCATGGGATCCATGTCTCAATTTGGACATGTTTC-3’; reverse: 5’-ACCGATGATACGAACGAGCTCTTAGACAACGCTTGCCTCAGCC-3’) were designed to amplify *AgPDS* gene from ‘Jinnan Shiqin’ cDNA using the high fidelity Phusion DNA polymerase (PrimeStar Max DNA polymerase; Takara, Dalian, China). The PCR products were purified and ligated into the pCAMBIA-1301 vector and then transformed into *E. coli* DH5α. The full-length of *AgPDS* was confirmed by Sanger sequencing (General Biotech, Anhui, China).

**Construction of CRISPR/Cas9 vector**

The target sites used for *AgPDS* gene editing were designed by the online software CRISPR-GE (http:// skl.scau.edu.cn/targetdesign/) ^6^. Four output target sites (20-bp gRNA sequence) located in the 5’-flanking region of exon were chosen to design sgRNA sequences. In order to reduce the off-target efficiency, the GC content of the designed target sites was not less than 40%. The 20-bp sequence had tri-nucleotide PAM (protospacer adjacent motif) sequence, i.e., 5’-TGG-3’ on its 3’ end. Four sgRNAs expression cassettes containing target site sequences driven by AtU3b, AtU3d, AtU6-1 and AtU6-29 promoters, respectively, were designed according to a previous report ^7^. The sgRNAs expression cassettes were inserted into the binary expression vector 2300GN-Ubi-Cas9 (a modified pYLCRISPR/Cas9Pubi-H vector, stored at the State Key Laboratory of Crop Genetics and Germplasm Enhancement, Nanjing Agricultural University; Fig. S2). The constructed recombinant vector contains Cas9 nuclease sequence, named *pCas9-sgRNA-AgPDS*.

***Agrobacterium*-mediated transformation of celery**

The recombinant plasmid *pCas9-sgRNA-AgPDS* was transferred into *Agrobacterium* *tumefaciens* strain GV3101 *via* electroporation, and then used to infect the explants of ‘Jinnan Shiqin’ that pre-cultured on Gamborg B5 solid medium within 2,4-D and KT (2:0.5) for about 12 days. To prepare the *A. tumefaciens* infection solution, a single colony carrying *pCas9-sgRNA-AgPDS* was picked and put in a 10 mL starter culture of YEB^+^ (YEB containing 100 mg/L rifampicin, 50 mg/L kanamycin and 50 mg/L gentamicin) and cultured in an incubator shaker (220 rpm) overnight at 28 ^o^C. Next, 1mL of the starter culture was transferred to 40 mL YEB^+^ and cultured until OD_600_ reached 0.5. The culture was centrifuged and the supernatant was removed. The pellets were resuspended using Gamborg B5 medium with 200 µM acetosyringone (pH 5.2) to reach the final OD_600_ = 0.4 and incubated on an incubator shaker (230 rpm) for 1 h. For infection, pre-cultured explants were infected for 20 min and then transferred to the Gamborg B5 solid medium containing 200 µM acetosyringone. After 2 days of co-cultivation at 25 °C in the dark, the explants were rinsed in sterile water and transferred to the selection medium supplemented with 2 mg/L 2.4-D, 0.5 mg/L KT, 300 mg/L carbenicillin and 50 mg/L kanamycin, which was renewed every 3 weeks. Generated celery calli was transferred to Gamborg B5 medium containing carbenicillin and kanamycin until regenerated plants are obtained.

**DNA extraction and mutation detection**

The genome DNA of putative *AgPDS* gene-editing mutant was extracted and purified using the Plant Genomic DNA Extraction Kit (Proteinssci, Shanghai, China), according to the operating instructions. The positive transformants were screened using *Cas9*-specific primers (Table S2, cas9-dF/dR). Then, the genomic regions of four target sites of *AgPDS* were PCR amplified using two pairs of primers, cut-F1/R2 and cut-F2/R1 (Table S2), that flank two sgRNA target sites each. The amplified products were used for direct sequencing (Sanger sequencing) to identify the mutation types. For the mutations that generated superimposed sequence chromatograms from direct sequencing, the 2,524 bp fragment of *AgPDS* that contains four targets were cloned into the pGBKT7 vector (Takara, Dalian, China) using the specific primers (F1-BK and R1-BK) and transformed into *E. coli* (DH5α) followed by sequencing individual subclones. The allelic mutations in transgenic lines were identified based on a single subclone that sequenced.

**References for Supplementary Materials and Methods**

1. Murashige, T., Skoog, F. A revised medium for rapid growth and bioassays with tobacco tissue cultures. *Plant Physiol.* **15**, 473-497 (1962).
2. Gamborg, O.L., Miller, R.A. & Ojima, K. Nutrient requirement of suspension cultures of soybean root cells. *Exp. Cell Res.* **50**, 151-158 (1968).
3. Li, M.Y. et al. The genome sequence of celery (*Apium graveolens* L.), an important leaf vegetable crop rich in apigenin in the Apiaceae family. *Hortic. Res.* **7**, 9 (2020).
4. Song, X.M. et al. The celery genome sequence reveals sequential paleo-polyploidizations, karyotype evolution and resistance gene reduction in apiales. *Plant Biotechnol. J.* **19**, 731-744 (2021).
5. Feng, K. et al. CeleryDB: a genomic database for celery. *Database (Oxford)* **2018**, bay070 (2018).
6. Xie, X.R. et al. CRISPR-GE: A convenient software toolkit for CRISPR-based genome editing. *Mol. Plant* **10**, 1246-1249 (2017).
7. Xu, Z.S., Feng, K. & Xiong, A.S. CRISPR/Cas9-mediated multiply targeted mutagenesis in orange and purple carrot plants. *Mol. Biotechnol.* **61**, 191-199 (2019).

**Supplementary Figures**

ATGTCTCAATTTGGACATGTTTCTTCTATCATTACACAAATTAATAATAATTTTAATAATAATAATTTCAATCTTCTCAAATTTCAATCTTCCCCCCTTTCATTTTCTGCTGCTACTCTCTCCACCTCCCTTAAATTCAACACTTCTTCTCTTTCCAAATTTACGCGCACTCGCCTGCTTAAGGTCAATTTTCTTATTTCCCAATTGTACTCTTTTTTGTTTTTGTATTTCTCCCCCCTTCCTCATTTAACCTTGAATGTTGCTCATACAGGTGTCTTGCGTCGACTATCCCAGGCCGGATATCGACAACACTCTTCCTTTTTTAGAAGCTGCTTACCTATCGTCTTTTTTTTCCACTGCTTCTCGTCCGTCTAAGCCATTAAATGTCGTAATTGCTGGTGCAGGTTAGTCCGTCTTTCTTTTTTCTTATATATTTATCCCATGCTCCACTTGCTCCTCTTTAGTACTATATAATTCTCTATTTAACAAGGAATGTACCAAACAAGCATACTACTAGCGCCCTAAACGATTTCAAACTTGGAAAATATACCATATACGATATATCCAATATGTCCTTACATGTACTTCGCAACTATGGTCACTATACTTAGGTCCAACACAAGTTCATGATGAGTTATTCACCAGCTTAAGTACACAAATTTTGATTAGCTAATAAAGGAATGTGGAGATATATTATATTCTATTAAATGTGCATATACATAATAACATGTCGTGCTTGCATTTTGGACGAGCTTGTGGTGTAAGTCGTGCTCTTGAATGCAACCTCCTTTCAGGTTTGGCTGGATTATCTACTGCAAAATATTTGGCAGATGCTGGTCACAAGCCCATATTGTTGGAAGCAAGAGATGTTCTTGGTGGAAAGGTTGGATCATAACTTCCCTCCTTTCACTTGCTAAACTATCCCGATGTTTCCACTGCATATGGAATAACAACAACCCTGCCTTTACTGAATTTGTGGAAATATCTTTTCCCTAACGAGAATAGTTTGCTTTTAGGTGTATTTTAACTTGATGATCTCGTTTAAGGATCATGTCTTATTAAAATTCATTTGATTAATTTGCTTCCTGCATTAATTGACTTGAGTTAATAAGACAAAAGAGCACCCTATGAATTTCTGTGAACACCTTGATTGATAATTCTGCTTCTGCTTATATCTCTGTATATTCAGTTTTCATTTTCTCATGAAGAATGTTTTACTTCATAAGGTGGCTGCATGGAAAGATGATGATGGAGACTGGTACGAGACTGGATTGCACATTTTCTGTGAGTTTTAGAGACTTAAATAATTAACTTTAATGATTTTTAAATATGGTTATTGTTCATTTCTGTCGCTTTAATAGTAATATATAGTTGACGTACTCTTAATGCAAGTGACAGTCTTTGATCACAATGTTTCTTTATATTTTCATGCACATATTACTTCGGTTTCCAGTATATTTCTATTAACCTTGTGTAAATTTGATTGAATTCTTCAAAAAAATTGTCTTGGCAGGTTTTATGCTATATAAATAGTACTTCCTTTGAATTCTTCAAATTAGTGTTTTGGCAGGTTTTCTGCTATATAAAAGTACTTCTGTATGACAAACAAAGAGCCCGTTTTTCATTTTTTTCCCCCATAAACATGATACATTGATTGTACACAAATTACCTCTCAGAATAGGAGTTCTTTAGCTTCCAATCAGTGATCGAAGAGTTTGTGATGTCAAACTTGAGGTTCTTATCTTCTTTTTTCCAAATAAAATTCCATCGTTTGTTATTTTCATTGCAAATGAAACTTTTTGCGTAATGGAGGGATTNACTATTAAAGCAAAGGATGGCTAGGTATTGAAGTTATAAGACAGCTTTGTTCAGAAAAGGAACAGGAACGTACAGTATTGAATTAATAATTATGTTGGTCAAGAATCAAGATCCCACCATAATGCATGACTATTTATGTAAAGCATGAGTCGTAACAAAGTTAGAGTACTGATGTTTCACAAAGTTTGATGGTTCTTTTTCTTTATCCAAGTTGGGGCTTACCCAAATGTTCAGAACCTGTTTGGGGAACTAGGAATTGACAACCGATTGCAATGGAAGGAGCATTCTATGATATTTGCTATGCCTAACAAGCCTGGGGAATTTAGTCGATTTGATTTTCCGGAAGTTCTACCTGCACCACTAAATGGCAAGTTTACTGTTATTATTTATTTATCATGATTAGATATTTAGATGTTTGATGTTTCCTGTCTCCTATCAATGATCGATTTTCAGTACGAGATTGTGCTACCAAAGTATACAGTTTCCTAAAAAAAATTCAAATTTATGTTACCCTTGTTACTTGTCCGGTGTAACTTGGTTTTCTCAGCCTATTTAACATCTTAACATTACTGCACAGGAATATGGGCTATCTTAAGGAATAATGAAATGCTAACATGGCCTGAGAAGATCAAGTTTGCATTGGGCCTCTTGCCAGCAATAATTGGTGGGCAAGCCTATGTGGAGGCGCAAGATGGTTTAAGTGTCCAAGATTGGATGAGAAAGCAAGTATGAATTCTTTAGTCATGTATTCTAGCTGACCATCACAACAAGTAGATCATGCAGCAGTTAATGAGTCTTGTATAATTTGTTCCACTTCCTCCAATTACAGCAGATAATGAGCTATTTCTTAGATTTGGCTTGCATCATATGTACACAAAAAAGTTGTCCAAGTAGACTCATATTTTTTTCTGTTTTGTACATAAAGTTGTTTAGACTCATCTTAAACTCATCATTTCTCTATCATTTTTCAGGGCATACCTGATAGGGTTACAACTGAGGTTTTTGTTGCCATGTCAAAGTCGCTAAACTTTATTAATCCAGATGAACTTTCTATGCAATGTGTATTGATTGCTTTGAACCGATTTCTTCAGGTACAACCACTATCTTTATTTGATAGGAATTATTAAACCTGTGCTACTTCAGGGACTTGGCAAATTACATGAATCTGGGAGTCCCTTGAGGTTCCAAATATATCTACATCTCTATATATGTCCAATTTCATACATAGATAAATGGGTATACTTTTTACTATCCCCCGCCCACATCTTTTTAATTAAAAGAGGTTGGCGGGCCCTTGTACCCTTTTATTCTTATTTTTGTGGTAGAATAGTCAATGAAGCTGTTTAACCAGTGTCTCCACTTTTCTATTGACATATAGGAAAACTTTTTTCCTTATTAATAGGAGGAAATTTATAGTAATTCTTAACGGAATAGACTTAGTACTTTCCTGATTGACTTTATGGATACTGTTTACTATGCATGTTGTTTCTCTTATAAAGAAATAATCTTTATGTTGGTACTAGTCGCTTTACACTCTTCATCTATGTGTGTAATGTCGTATTCACAATACTCATTACAATGAAATAAACCCTTAGAAAATTCATTTTCACTAGTAACTGAGATCTAAGAGATTATAACTTAAAGATATATCATTTATCTGACTAGAATGCGCTTAAAATAGTTGTGGAAAGTGTATTAAGGGCATCTCCAATGGCTGCTTTAAAAGCCCCCTGACCTTTATATTACTTCATGTTTGAAGTTAAAGTCTCTAAAATGTATCTCCAATGATGGTCAATATCATCTTCATTTTTGAAGTAATATACTGTACAGCTCTATATTTGAAGACGCACTGTACATATAAAGCAAAGCATCACCGGCTGACACATGGTCCATTCCACTTCCTGTATAATAACATGCCATAGTTAGTGTTACACGTGTGTATGTTTATAGTTTTATATGTATATAAAATGTGTTTGAAGTAAAATTTGAAGGAATGGTTGGAGAAGAAATAGTGATTTGAAGTGAAAAAGGTGAAAATTTAAAGATAGAGTTGTTTTAAAGTAGAGTAACGGTCGGAGAAGGCCTTAGGATTTTTGGTGTTAAAATTGAGGAATACGCAAGATGTTGCTTTTGCATTGAATGGTTAAATAAATATGCTCGAAACATGACAGGAGAAGCATGGTTCAAAGATGGCTTTCTTGGATGGAAGTCCTCCAGAAAGACTTTGCATGCCTATAGTTGATCACATACAGTCACTGGGTGGTGAAGTTCATCTCAATTCACGAGTACAGAAGATCTCTTTGAATAAAGATCATACTGTTAAGAGTCTATTACTAACCAATGGGAAGGTTATCGAAGCAGATGCATATGTTATTGCTGCTCCAGGTGATTTCCTATTTATTCACTTTCATATATAGATAAGTTCATATTTTCTAATTTTCTAATTTCGTTGATTCTTTTTTTTAAATTAGTTGATATTCTAAAGCTACTCGTGCCTGAAGAGTGGAGAGAGATTCCATACTTCAAGAAGTTGGATAAATTAGTTGGAGTTCCAGTAATCAATGTTCACATATGGTTAGTTATCACAATTTTTTGAGATTTTCATTATAATGAATTTTCCGTTGACTGCATCTTTTCATCATTGGTGCTGTTCATAATTATTCTTTCTGTGCATTTCTACAGTCTCCAGTTAAATTGTAAAATTGATTGCTTACTGATAATCTGTAGTCAACAAACTAAATTTGCTATACTCTCGATGTCTTGTTGACATGAATTAATATCACTTTCAGGTTCGACAGGAAACTGAAGAACACATATGATCATCTACTTTTCAGCAGGTGCCCTTTATTAAATAAACATATTATTTTTTATATCATCTTGTGAAGCACATGTCTTAAAAGCTCAAACTTTTTTGCCATTTCTCAAATATTCTAATGAAGAATTAAATTGAACACGTGTGATGGTTGTTTCCGTAGTATTGTTACTTATAGTCTTTGTTTTGCTTCATTATTTGTAAATGCACGCATGAGATGCTTTTGCACTTGTCTTTTTCTTATTTTTTGGGGGTGGGGAGGGGGGTTGTAAGAAGGTGAAAGAAGCCTCTTGCATGACATTTGCCAGAGTTCTATTAGTTTCTAAGAATATGAAGTAGCAGACGTTGCGACTTTTAATACTACAGGACCAAGGGTCCGCCTTCATTACATTTGTGTCATGGTAATTTTGGTCCTTATTCTATACCCAGCTTCCATATTCATCTGTTCTCTGATTTTCTGCAGAAGCTCACTTCTCAGCGTATATGCTGATATGTCCGTAACTTGTAAGGTAATTTAACATTATTTCTCTCTAAAATTTCCTGCTTCATATCTGTAGAAATTGGTACTACTTACCATATTGTGTTTTTCTCAAATGAACAATTTACTTTAAGATTCTCTGGTGTAGGCTCAAATCCTGTTCACTTTCCCGCTGCTGATAGTACAGAACTAATCATTCCCTCTGGCATGCTAAAAGTAAAGAACATAAATAAATCCACATAACTTGAATTTAAGGATTATATTATCTAGATTAATAAATCGGTTTTGAAAGAGAGTGAGTTGAACAAATTTAAAAACGAAAACAGTCCAATTCTTAACAAAATACAGTCAGCTAAAGGATGTGCCTTAAAAAGTATTGTATTGTTTTGTACAGGAATATTACGACCCAAATAAGTCCATGCTGGAGTTGGTTTTTGCACCTGCAGAAGAATGGATTTCACGCAGTGACTCTGACATCATTGATGCAACGATGAATGAACTGGCCAGACTATTTCCTGATGAGATTGCTGCTGATCAGAGCAAAGCAAAAATACTGAAGTACCATGTTGTTAAAACACCAAGGTCAGCAGTGCAATTTCTTACTATCATAGTGCTATATTGTCGATTGCAAAAATATATTGGTATACACCAAACTTAAGTTAGATATAAATTAATTAGTATGCATTATGCGCCGAGTATGAGGTATAACCTGGTGTGGTATGTTAAATCTCCTTCAGTTGTACTGATTTGCATATCAGATCCGATGATTAATATATAATTATGAACCTTTAAAGAATAAATCCGCTTCTATCATTTGTTGCATTACCATTTACATTTCCTATGATTCAGGTCTGTTTATAAAACTATACCAGACTGTGAACCCTGCCGCCCTTTGCAAAAATCTCCCATAGAAGGTTTTTATTTAGCTGGTGATTACACAAAACAGAGGTATCTGGCTTCAATGGAGGGTGCTGTCCTCTCGGGAAAGCTTTGTGCTCAAGCAATATTGCAGGTAAAATTATGGTTATTAGTTATTACTATTGACAGATGTATTCCACATTTACATCACTTTGTCGGTCATAGTTTATTTCATTTTTCACTAAATATTCTGATCTTTTAGTTCAAGGCTCTCAACAAAAAACCTAGTAATTTGCACCAATATTTTAAAATTTCTGGTTGAAGTCCTGTTCGTCAAAAATTTAACGGAGTTAATATATCTCAAGTTGTATCTTAGAATCAGTAAAGAATTTTTGTCTGAAATTCAGCGGAGTCCGCCATTAGAATCAAAATAGAGTTTTTTTAATTCCATTATCACCTACTCTTTGATCATTTTTATGACTTTTCAACCTCTCCTTGACAAACCATCCTCTTCCCTAAAACTCTAACCGCAAAACTTAACAGTCTAACCCTCCTCTTATGCACAAACCACGCCCCCATAATCCGATCTCTCACAGTCACCTACCTCGAAACTACAGACAATCTCAGGAAGTACTTTCTTATTTCCACTTTGATTACACGAATTTAATTGTCCCACCACCATCAGGAAGTATTAAAATAGAAAAATTGCTATCACATGGTTCTAGGAACTTATTAGTTTGACCACTAAATTCAAATATGTTTTAATTTTAATGGTGGATTCTGGCAAAAATTCATGATTTTCCAACTTAAAAATCTGTTTTAGGTTTCTTAAGGGACTTGAACCAAAACCCCTTATAATTGTGTGACTACTAAATCAGTTATCTTGTTTCTATGAACATACNTTTTAATGGTGGATTCTGGCAAAAATTCATGATTTTCCAACTTAAAAATCTGTTTTAGGTTTCTGAAGGGACTTGAACCAAAACCCCTTATAATTGTGTGACTACTAAATCAGTTATCTTGTTTCTATGAACATACAAGTAATATTCACGTATTAAATTATATCTTACTCAGCTGTCACTCACATGCCAACGTCAGTGAAAATATGTTGACTACCGGAAGGACCTCAATCAGATTTTTTTTAAATAAGGAGATGAACGACTGGGTTTATTGTTCAGGGACATGAACCAGAAAGTCTGAATAGTTTAGTGATTAATGGATTAATAATCCCTAATATACATTGTGGAGAGAATTTAAACTCGAAAGTATAATAAAATGAAATTATAGCCCGTTCACCTTTGTCCTCTATTTACGTCTTATGTATTAAATTATGCAGGATCATGAGTCGCTGCTTTCTCGCAGGAAGCATGTGCTGGCTGAGGCAAGCGTTGTCTAA

**Fig. S1 The sequence of *AgPDS* gene from celery cv. ‘Jinan Shiqin’.**

Introns (black) and exons (blue) are represented in black and blue, respectively.


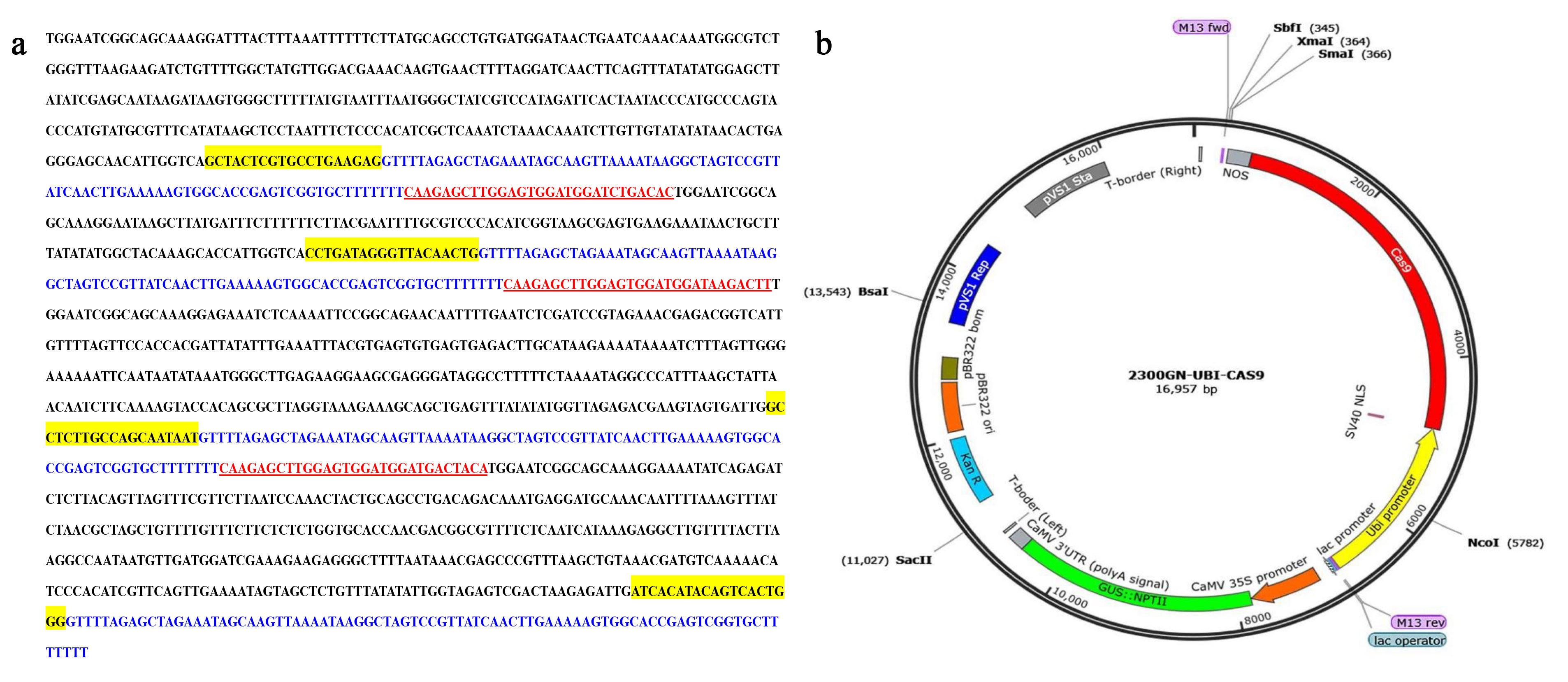


**Fig. S2 The sequence of sgRNA expression cassette and map of the Cas9/sgRNA vector used in this study.**

(**a**) The design of the vector construction used for *AgPDS*-knockout. Four target sites, highlight with yellow; Conservative structural sequence, blue colored; The promoter sequences of AtU3b, AtU3d, AtU6-1 and AtU6-19, black colored. (**b**) The vector map of 2300GN-Ubi-Cas9 was cited from Li et al., 2022.


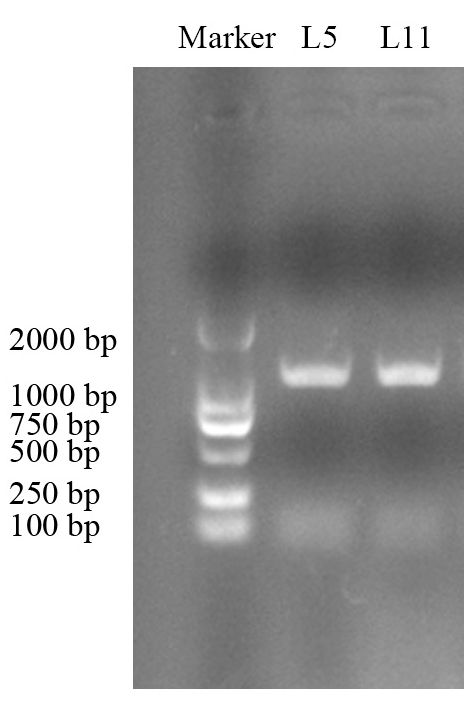


**Fig. S3 PCR amplification for identifying the presence of *Cas9* in two *AgPDS*-knockout lines (lines 5 and 11).**

The PCR products were amplified with specific primers cas9-dF and cas9-dR (listed in Table S2).


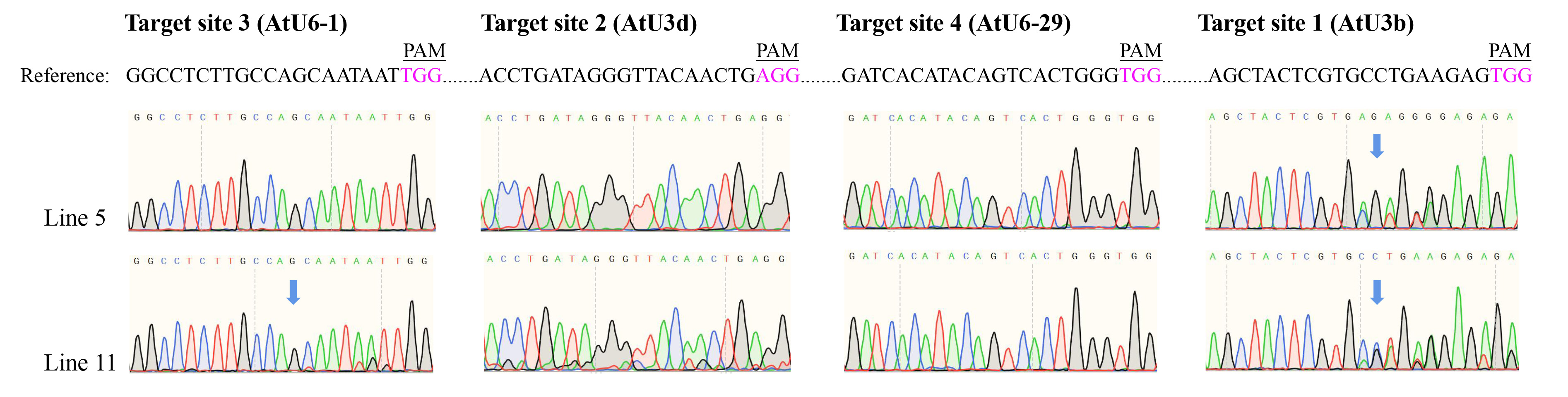


**Fig. S4 The chromatograms from direct sequencing of two independent *AgPDS* mutant plants.**

The blue arrow points out the superimposed sequence chromatograms, indicating that a potential mutation is occurred at the corresponding target site.

**Supplementary Tables**

**Table S1 The induction rate and differentiation rate of ‘Jinnan Shiqin’ calli induced by different hormone combinations.**

| 2,4-D (mg/L) | 0.5 | 0.6 | 0.5 | 1.0 | 1.0 | 2.0 | 2.0 |
| --- | --- | --- | --- | --- | --- | --- | --- |
| KT (mg/L) | 0.6 | 0.8 | 1.0 | 0.5 | 1.0 | 0.5 | 1.0 |
| induction rate | 75.8% | 61.7% | 77.2% | 98.3% | 97.4% | 95% | 87.3% |
| differentiation rate | 63.6% | 52.9% | 58.8% | 77.1% | 33.33% | 78.5% | 71.8% |

**Table S2 List of primers used in this study.**

| Primer name | Sequence |
| --- | --- |
| cas9-dF | CTACTTCTTTTTCTTAGCCT |
| cas9-dR | CAAGGCCACCGCCAAGTACT |
| cut-F1 | ATGATATTCGCTATGCCTAACA |
| cut-F2 | GACAGGAGAAGCATGGTTCAA |
| cut-R1 | TGTTCTTCAATTTCCTGTCGAAC |
| cut-R2 | CTGAAGAAATCGGTTCAAAGCA |
| F1-BK | TGGCCATGGAGGCCGAATTCATGATATTCGCTATGCCTAACA |
| R1-BK | TGCGGCCGCTGCAGGTCGACTGTTCTTCAATTTCCTGTCGAAC |
